# Current News

**Published:** 1904-01

**Authors:** 


### Current News.

### FORM OF AGREEMENT OF THE STATE BOARD OF NEW JERSEY.

AGREEMENT entered into this ———— day of ————, 190 , between the State Board of Registration and Examination in Dentistry of New Jersey, Members of the National Association of Dental Examiners, and the State Board of Examiners of the State of ————, also members of the same body.

*Witnesseth,* The party of the first part hereby agrees under the terms of the resolution passed at Asheville, to exchange with the State Board of Examiners representing the State of ———— licenses to practise dentistry, and to issue license to the candidate presenting credentials properly filled out, accompanied with fee such as the laws of each State require.

*In witness thereof* the president and secretary have signed their respective names and seals in duplicate.

### ASHEVILLE RESOLUTION.

"*Resolved,* That an interchange of license to practise dentistry be, and is hereby recommended to be, granted by the various State Boards, on the following specific conditions:

"Any dentist who has been in legal practice for five years or more, and is a reputable dentist of good moral character, and who is desirous of making a change of residence into another State, may apply to the Examining Board of the State in which he resides for a new certificate, which shall attest to his moral character and professional attainments, and said certificate, if granted, shall be deposited with the Examining Board of the State in which he proposes to reside, and the said Board, in exchange thereof, may grant him a license to practise dentistry."

————————————

*President State Board of* ———— [SEAL]

————————————

*President State Board of* ———— [SEAL]

————————————

*Secretary State Board of* ———— [SEAL]

————————————

*Secretary State Board of* ———— [SEAL]

### FLORIDA STATE DENTAL SOCIETY.

The twenty-first annual meeting of the Florida State Dental Society will be held at Atlantic Beach, May 25, 1904. Following are the officers and standing and special committees for 1903–4:

President, Dr. J. Edward Chace, Ocala, Fla.; First Vice-President, Dr. A. B. Whitman, Orlando, Fla.; Second Vice-President, —Dr. R. L. McMullen, Clear Water, Fla.; Secretary, Dr. D. D. Beekman, Daytona, Fla.; Treasurer, Dr. F. B. Hannah, Jr., Jacksonville, Fla.

*Executive Committee.*—Dr. Jas. W. Simpson, Kissimmee, Fla.; Dr. Jas. E. Chace, Jacksonville, Fla.; Dr. A. J. Hannah, Umatilla, Fla.; Dr. G. Enloe, Miami, Fla.; Dr. A. B. Stevens, Orlando, Fla.

*Operative Dentistry.*—Dr. J. D. L. Tench, Gainesville, Chairman; Dr. Edith R. Brush, Daytona, Secretary.

*Mechanical Dentistry.*—Dr. F. E. Buck, Jacksonville, Chairman; Dr. Jas. W. Simpson, Kissimmee, Secretary.

*Physiology and Etiology.*—Dr. W. E. Driscoll, Braidentown, Chairman; Dr. Henri Letord, Orlando, Secretary.

*Dental Education and Literature.*—Dr. W. G. Mason, Tampa, Chairman; Dr. W. S. Taylor, De Land, Secretary.

*Essays and Voluntary Papers.*—Dr. C. H. Frink, Fernandina, Chairman; Dr. R. L. McMullen, Clear Water, Secretary.

*Pathology and Surgery.*—Dr. L. C. Elkins, St. Augustine, Chairman; Dr. G. Enloe, Miami, Secretary.

*Dental Histology and Microscopy.*—Dr. T. J. Welch, Pensacola, Chairman; Dr. R. M. Mason, Sanford; Secretary.

*Dental Chemistry and Therapeutics.*—Dr. A. B. Whitman, Orlando, Chairman; Dr. E. A. Law, Bartow, Secretary.

*Clinics.*—Dr. C. C. Collins, Atlanta, Ga., Chairman; Dr. W. McL. Dancy, Jacksonville, Secretary.

*Arrangements.*—Dr. Jas. Chace, Jacksonville, Chairman; Dr. E. M. Sanderson, Jacksonville, Secretary; Dr. F. E. Buck, Jacksonville.

*To investigate Illegal Practice.*—Dr. W. G. Mason, Tampa, Chairman; Dr. T. J. Welch, Pensacola, Secretary.

*To revise the Constitution and By-Laws.*—Dr. C. F. Kemp, Key West, Chairman; Dr. J. E. Chace, Jacksonville, Secretary; Dr. L. C. Elkins, St. Augustine.

The latter committee was appointed to draft a new Constitution and By-Laws more in accord with present needs. Members of the society are urged to make such suggestions to the chairman as may occur to them.

It is urgently requested that all members, whether on a committee or not, will contribute papers or clinics, communicating their willingness to the chairman of the committee to which they wish to be assigned.

Any suggestions which will help to make our next meeting a success will be gladly received by the President or Secretary.

J. Edward Chace, D.D.S., *President.*
D. D. Beekman, D.D.S., *Secretary.*

---

### THE INTERSTATE DENTAL FRATERNITY OF THE UNITED STATES AND CANADA.

At the Annual Meeting of the Interstate Dental Fraternity, held at Asheville, N. C., on July 29, 1903, the following officers were elected for the ensuing year:

Vice-Presidents: For Arkansas, C. Richardson, D.D.S., Fayetteville; for California, H. P. Carleton, D.D.S., San Francisco; for Connecticut, James McManus, D.D.S., Hartford; for District of Columbia, Emory C. Bryant, D.D.S., Washington; for Illinois, Hart J. Goslee, D.D.S., Chicago; for Indiana, George E. Hunt, D.D.S., Indianapolis; for Kansas, George A. Esterly, D.D.S., Lawrence; for Louisiana, Edward Kells, D.D.S., New Orleans; for Maryland, B. Holly Smith, D.D.S., Baltimore; for Massachusetts, John F. Dowsley, D.D.S., Boston; for Minnesota, Frank E. Moody, D.D.S., Minneapolis; for Missouri, Burton Lee Thorpe, D.D.S., St. Louis; for New Jersey, Charles S. Stockton, D.D.S., Newark; for New York, F. C. Walker, D.D.S., Brooklyn; for North Carolina, J. A. Gorman, D.D.S., Asheville; for Ohio, Henry Barnes, M.D., Cleveland; for Pennsylvania, I. N. Broomell, D.D.S., Philadelphia; for Rhode Island, Dennis F. Keefe, D.D.S., Providence; for Wisconsin, H. L. Banshaf, D.D.S., Milwaukee.

R. M. Sanger,
*Secretary.*

